# A Novel Assessment of Flexibility by Microcirculatory Signals

**DOI:** 10.3390/s140100478

**Published:** 2013-12-30

**Authors:** Jian-Guo Bau, Taipau Chia, Yu-Fang Chung, Kun-Hao Chen, Shyi-Kuen Wu

**Affiliations:** 1 Department of Biomedical Engineering, Hungkuang University, No. 1018, Sec. 6, Taiwan Boulevard, Taichung City 43302, Taiwan; E-Mail: baujg@sunrise.hk.edu.tw; 2 Department of Safety, Health and Environmental Engineering, Hungkuang University, No. 1018, Sec. 6, Taiwan Boulevard, Taichung City 43302, Taiwan; E-Mail: tpchia@sunrise.hk.edu.tw; 3 Department of Electrical Engineering, Tunghai University, No. 1727 Sec. 4, Taiwan Boulevard, Taichung City 40704, Taiwan; E-Mails: yfchung@thu.edu.tw (Y.-F.C.); a46825a5@gmail.com (K.-H.C.); 4 Department of Physical Therapy, Hungkuang University, No. 1018, Sec. 6, Taiwan Boulevard, Taichung City 43302, Taiwan

**Keywords:** flexibility, Laser-Doppler Flowmetry, stretching, chronic venous insufficiency, gastrocnemius muscle, perfusion, pulsatile

## Abstract

Flexibility testing is one of the most important fitness assessments. It is generally evaluated by measuring the range of motion (RoM) of body segments around a joint center. This study presents a novel assessment of flexibility in the microcirculatory aspect. Eighteen college students were recruited for the flexibility assessment. The flexibility of the leg was defined according to the angle of active ankle dorsiflexion measured by goniometry. Six legs were excluded, and the remaining thirty legs were categorized into two groups, group H (*n* = 15 with higher flexibility) and group L (*n* = 15 with lower flexibility), according to their RoM. The microcirculatory signals of the gastrocnemius muscle on the belly were monitored by using Laser-Doppler Flowmetry (LDF) with a noninvasive skin probe. Three indices of nonpulsatile component (DC), pulsatile component (AC) and perfusion pulsatility (PP) were defined from the LDF signals after signal processing. The results revealed that both the DC and AC values of the group H that demonstrated higher stability underwent muscle stretching. In contrast, these indices of group L had interferences and became unstable during muscle stretching. The PP value of group H was a little higher than that of group L. These primary findings help us to understand the microcirculatory physiology of flexibility, and warrant further investigations for use of non-invasive LDF techniques in the assessment of flexibility.

## 1. Introduction

Physical health is usually assessed according to some health-related fitness components, like a morphological component, a muscular component, a motor component, a cardiorespiratory component and a metabolic component [1,2]. Among these components, the assessment of muscular components provides health information about muscles or muscle groups. The conventional muscular assessment, which is the essential requirement for the diagnosis of musculoskeletal disorders in rehabilitation and sports medicine, includes muscle strength, muscular endurance and explosive strength. Although flexibility is not categorized as a muscular component, it provides the physiological information about muscles and is another health index specifically designed for athletic performance and the capacity to carry out the daily activities.

As muscular flexibility is an important aspect of health, muscle tightness is frequently postulated as an intrinsic risk factor in the development of a common muscular dysfunction. This disorder is often accompanied by pain, muscle weakness, and restricted range of motion. Limited joint range of motion has been regarded as a predisposing factor in a number of lower limb injuries, including muscle strains, stress fractures [3], and patellofemoral syndrome [4]. Maintaining normal muscle length requires regular stretching to prevent muscle stiffness and benefit from the decreased risk of musculoskeletal injuries and enhance exercise performance [5,6].

The typical flexibility tests, including *side bending* and *sit and reach*, are the evaluations related to whole body flexibility. The measurement of range of motion by goniometry performed around a joint center and surrounding body segments provides the regional flexibility. The calf muscle flexibility test is a simple indirect flexibility test, which usually requires a ruler or tape measure. The procedure is to ask a subject to stand flat footed the maximum distance away from the wall and also be able to bend the knee to touch the wall. The maximum distance from toe to the wall is the calf muscle flexibility. However, the variation in leg length can make comparisons between individuals misleading. Moseley proposed that the static stretching was used with the muscle in a relaxed position, and the flexibility assessment was made by measuring the distance from the starting position to the end of the movement, or stretch [7]. The indirect measurement consists of clinical examination of joint ranges, but this is subject to a number of systematic and random errors. Some factors must be taken into account when establishing muscle flexibility by the methods mentioned above, such as joint structure, ligaments, tendons, muscles, skin tissue, fat (or adipose) tissue, which may influence an individual's range of motion about a joint [8].

Little attention has been paid to the assessment of muscle flexibility from the microcirculatory aspect point of view, while vascular impairment is widely acknowledged as an important factor in acute and chronic muscle lesions. Recently, Otsuki investigated the changes in muscle blood perfusion and tissue oxygenation determined by non-invasive near infrared spectroscopy (NIRS) signals between subjects with different flexibility [9]. He concluded that the muscle blood flow and muscle oxygenation in ballet-trained subjects were less interfered with by passive muscle stretching than in untrained subjects. Another relative research also suggested that the vascular stability was essential for tissue health, while an instable microcirculatory supplement might further impair blood-tissue oxygen exchange and therefore caused the consequent impairment of tissue function [10]. In the studies of muscle physiology on office workers with low level, repetitive and static computer tasks by using Laser-Doppler Flowmetry (LDF), researchers found a significant association between the chronic musculoskeletal pain and trapezius vasodilatation [11–13]. This vasodilative characteristic was shown to be more sensitive than the muscle activity from the records by electromyography. Unfortunately, the tissue perfusion signals were determined by the single-fiber LDF technique with optic-fiber probe inserted invasively into the upper trapezius in these investigations, which therefore made it not practical for use in clinical applications.

Recently, a high power LDF with wide separation probe was developed to explore its potential for the assessment of deeper tissues in humans for non-invasive application [14]. Since the microvascular perfusion function may be associated with muscle flexibility, the aim of this study is to develop convenient indices for the assessment of muscle flexibility by analyzing the characteristics of blood perfusion determined by non-invasive LDF technique during different muscle stretching and relaxed states. After the signal processing with the modified beat-to-beat algorithm [15,16], the flexibility indices can be defined in participants with different flexibility levels of calf muscle.

## 2. Materials and Methods

### 2.1. Subjects

Eighteen college students (nine male, nine female), age 20–21 years were recruited for the flexibility assessment. All participants were informed the purpose of this study and assured confidentiality and anonymity, and their informed consent was obtained before the study. Potential participants were excluded when they had: (1) ankle or knee symptoms within 1 month prior to the enrollment; (2) arthritic or other inflammatory diseases; (3) bone pathology; (4) neurological system dysfunction; or (5) history of ankle trauma or surgery. Six legs were excluded, and the remaining thirty legs were categorized into two groups, group H (*n* = 15 with higher flexibility) and group L (*n* = 15 with lower flexibility), considering Moseley's criteria for leg flexibility [7]. The flexibility was defined according to the ankle RoM [8]. The participants in both groups were matched for the body weight and height (Table 1). Besides, the participants were requested to maintain a regular diet and get adequate sleep as well as to avoid vigorous-intensity physical activities one day before the experimental trial. They were also asked for not having any food or drink or exercise at least 1 h before each test and to refrain from alcoholic and caffeine-containing drinks on the trial day.

### 2.2. Instrumentation

**Figure 1.**
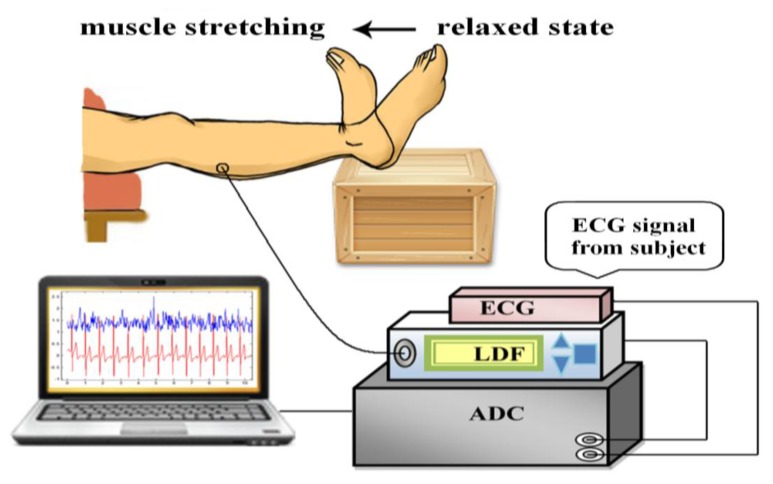
The measurement system, the location of the measurement site of microvascular perfusion, and active gastrocnemius muscle stretching with ankle dorsiflexion.

Figure 1 shows the measurement system applied in this investigation. The system measured the microcirculatory blood perfusion and electrocardiogram (ECG) from the participants simultaneously and synchronously. The acquired analog signals were sampled via an analog-to-digital converter (ADLINK, PCI-9111DG, Taipei, Taiwan) with a sampling rate of 1,024 Hz and then analyzed using a personal computer. The location of microcirculatory measurements was on the belly of gastrocnemius muscle at the posterior of the lower leg. The ECG signals in the lead II configuration were monitored by the bio-impedance amplifier (EBI100C, BIOPAC System, Goleta, CA, USA) with surface electrodes. The microcirculatory signal was detected using LDF (VMS-LDF1-HP, Moor Instruments, Axminster, Devon, UK) in a sampling frequency of 40 Hz and a skin probe (VP1-V2-HP) with optical fiber separation of 4 mm. A laser with the power of 20 mW and the wavelength of 785 nm was adopted in the applied LDF. LDF was calibrated before measurements using aqueous suspension of polystyrene latex particles. All of the measurements were conducted according to laser safety requirements (Class 3R per IEC 60825-1:2007).

### 2.3. Experimental Protocol

**Figure 2.**
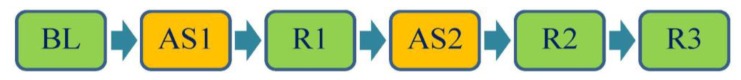
Six measurements in the experimental protocol schedule. Muscle was in relaxed states during BL, R1, R2 and R3, whereas AS1 and AS2 were the stretching states.

Before the experimental data collection, the participants stayed in the experimental environment with the temperature maintained at 26 ± 1 °C for at least 20 min and then supine on a comfortable couch with full support of relaxed lower extremities. The lower leg placed over the edge of couch and the foot was supported by a stool (Figure 1) during the whole trial. Furthermore, the measurement protocol was performed as shown in Figure 2. Six 1-min measurements were conducted sequentially with 1 min interval between measurements in each trial. After the first 1-min measurement of baseline (BL) taken in relaxed state, the participants actively stretched their gastrocnemius muscles by performing ankle dorsiflexion to the end range (Figure 1). The measurement in active stretching state (AS1) was taken simultaneously for 1 min. After AS1, one measurement with muscle relaxed state (R1) was conducted, which was followed by the fourth measurement with active stretching (AS2). Finally, two measurements (R2 and R3) with relaxed state were taken. After these measurements, the angle of ankle dorsiflexion range was assessed for each participant with a goniometer while the participants actively stretched their gastrocnemius muscle by performing ankle dorsiflexion to the end range (Figure 1) with the knee in extended positions. Three measurements were recorded for each leg and averaged to yield representative values. The active RoM of ankle ranged from 3.7 degrees to 28.3 degrees. The mean (SD) of the RoM for group H and group L were 20.3 (4.3) degrees and 10.8 (3.7) degrees respectively (*p* < 0.0001). Moseley suggested the lesser ranges of ankle dorsiflexion in knee extended indicated that the gastrocnemius muscles were tight and inflexible [7]. All the trials were performed within 8:30 AM–11:00 AM or 2:00 PM–4:30 PM to avoid the interference from fasting or postprandial effects, or fatigue from work.

To assess the consistency among LDF measurements conducted by multiple operators, a pilot inter-rater reliability test was performed on ten subjects before the trials. One minute baseline (in relaxed state) of LDF was taken for each subject by two operators, respectively.

### 2.4. Signal Analyses

**Figure 3.**
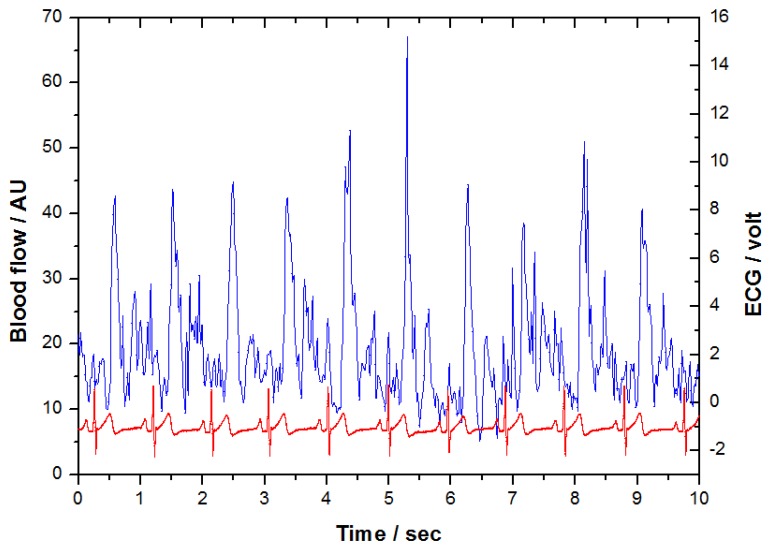
The typical blood flow signal determined by LDF (blue), plotted together with ECG signal (red).

Figure 3 shows the original LDF and ECG signals obtained from the measurement system synchronously. There were 61,440 samples (1,024 × 60) for each 1-min measurement with a sampling rate of 1,024 Hz. When inspecting two coherent signals in this Figure 3, the signal with distinctly repeated patterns is the electrocardiogram. The other one which does not have a specific pattern is the LDF signal. It can be appreciated the peaks of LDF signal appeared synchronously correlated with the R-peaks of ECG signal after a certain phase delay. In order to determine the length of the LDF signal of each beat, the “beat-to-beat” algorithm was applied [15–17]. According to this algorithm, the “waveform” of LDF signals in heart-beat frequency was derived. Based on the characteristics of microcirculatory waveform, three quantitative indices were developed respectively. The five steps of the signal processing are illustrated as follows:

#### 2.4.1. Signals Filtering

Both LDF and ECG signals were filtered by a digital high-pass with a cut-off frequency of 0.1 Hz to eliminate the respiratory activity and other baseline drift. After that, a low-pass filter with a cut-off frequency of 55 Hz was applied to avoiding the electrical noise from instruments.

#### 2.4.2. Segmentation of LDF Signal

When the ECG R-peak of each heartbeat was located, the period of each heartbeat was then determined. Because ECG and LDF signals were measured synchronously, the LDF signals were divided into the same length as each R-R interval of ECG. Therefore, the LDF signal between two R-peaks could be regarded as one perfusion pulse in a heartbeat to obtain N segments of perfusion pulse. N is the heartbeat number in the 1-min period of measurement. The value of N may be different for each subject.

#### 2.4.3. Normalization of LDF Segments

Though human's heart beats regularly most of the time, the period of each beat is not equivalent. The median of the N pulse periods was selected as the standard period of one heartbeat, and all LDF segments were normalized into the same period using a MATLAB “resample” algorithm (MathWorks, Natick, MA, USA).

#### 2.4.4. Derivation of Mean LDF Waveform

**Figure 4.**
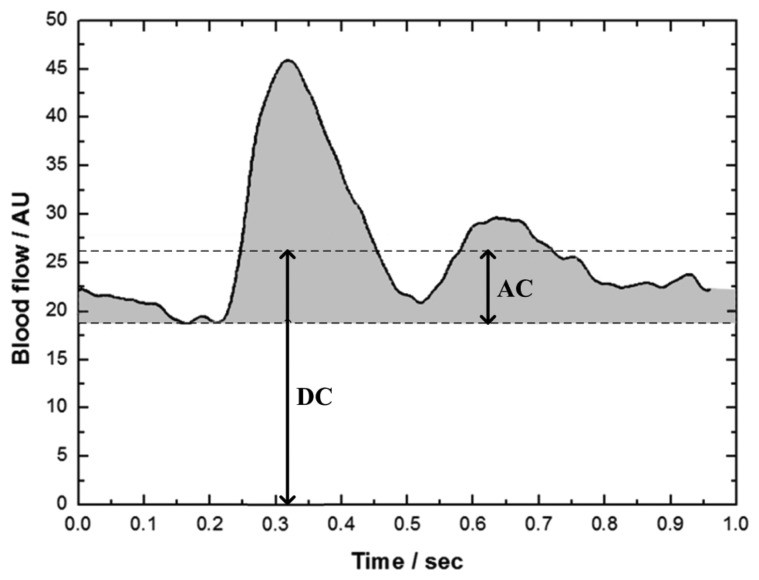
The definitions of DC and AC components of mean LDF waveform derived from segments of LDF signals.

Figure 4 shows the mean LDF waveform, which was the mean of the N LDF segments with the same period. This mean LDF waveform represented the variation of blood flow within one heartbeat interval. The zero of x-axis represented the time of occurrence of R-peak. The maximum of blood perfusion waveform located at about 0.2–0.3 s after R-peak.

#### 2.4.5. Index Calculation

Among the noninvasive methods for monitoring the perfusion in peripheral tissues, the peripheral perfusion index (PI) defined as the ratio of pulsatile to nonpulsatile components of photoplethysmographic waveform in pulse oximetry has been widely applied in clinical application [18,19]. Similarly, three indices of DC value, AC value and perfusion pulsatility (PP) were defined from the mean LDF waveform in the present study (Figure 4). DC value is the mean blood flow of the mean waveform, which is the blood perfusion in tissue for general clinical use. AC value is the mean of the pulsatile part (shadow area) of the mean waveform. PP value is defined as the ratio of AC value to DC value as shown in Equation (1):

(1) $$\text{PP}=\frac{\text{AC}}{\text{DC}}$$

### 2.5. Reliability Analyses and Statistics

In the pilot inter-rater reliability test, the mean values of 1-min LDF baseline taken by the different operators were calculated for each subject. The reliability was then evaluated by intraclass correlation coefficients (ICC) [20,21]. In the formal trial, the mean values of LDF signals over 20 s were calculated, giving three time periods for 1-min baseline measurements, to evaluate the reliability of the LDF measurement during resting conditions for both group H and group L. The reliability was also analyzed by ICC. The ICC was considered high above 0.8, moderate 0.6–0.8, and poor below 0.6.

All three indices of the LDF perfusion signals between group H and group L were compared to study the inter-group variation by using an independent *t*-test. In order to understand the effects of active muscle stretching on microcirculation, a paired-sample *t*-test was used to study the difference of LDF signals between two adjacent measurements with the muscle in different states. The differences were considered as significant when two-tailed significance level *p* < 0.05. The Statistical Package for Social Science (release 12.0, SPSS Inc., Chicago, IL, USA) was used for the statistical analysis.

## 3. Results

### 3.1. Reliability of Blood Flux between Operators and during Resting Conditions

LDF values monitored by different operators exhibited a consistent pattern, with ICC = 0.69. The resting values for LDF were also stable within the 1-min baseline. The reliability of LDF during baseline was high for the measurements in both groups, with ICC = 0.99 and ICC = 0.99 for group H and group L, respectively.

### 3.2. Flexibility Analyses

**Figure 5.**
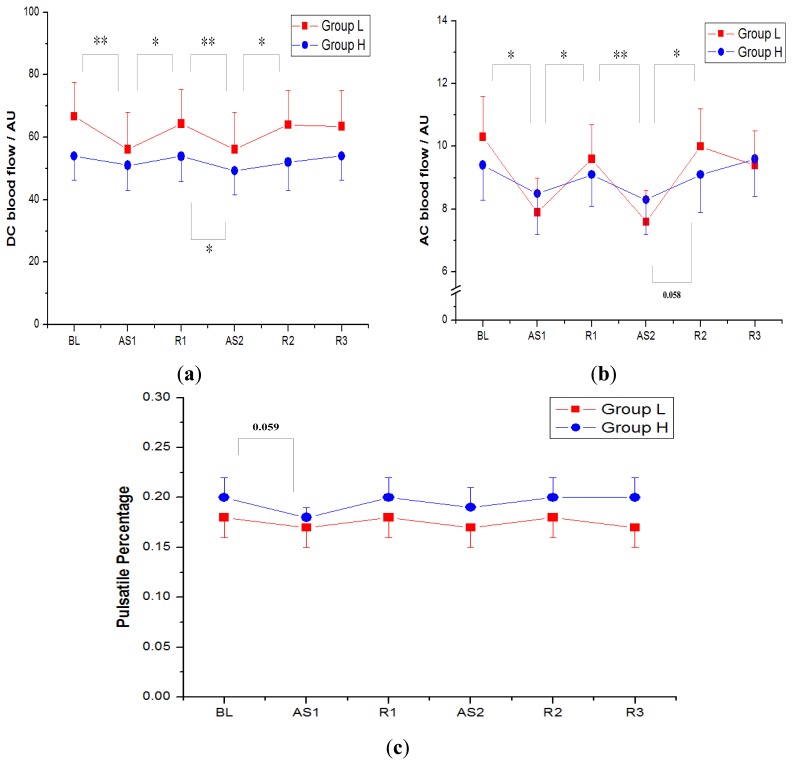
(**a**) The nonpulsatile (DC) component of the blood perfusion on gastrocnemius muscle in the higher flexibility group (group H: 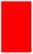) and the lower flexibility group (group L: 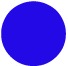); *n* = 16. (**b**) The pulsatile (AC) component of the blood perfusion on gastrocnemius muscle in both group H and group L. (**c**) The PP values (AC to DC ratio) of the blood perfusion in the two groups. * Statistical significant at a level <0.05, **<0.01.

Figure 5 shows DC (nonpulsatile component), AC (pulsatile component) and PP indices (AC to DC ratio) of the LDF perfusion signals monitored on gastrocnemius muscle in both group H and group L. The data are expressed as the mean ± 1SE. Along six sequential measurements (BL, AS1, R1, AS2, R2, R3), the DC values (mean ± SE) of group H appeared 54.0 ± 7.7 arbitrary units (AU), 51.0 ± 7.9 AU, 53.9 ± 8.1 AU, 49.3 ± 7.7 AU, 52.0 ± 8.9 AU and 54.0 ± 7.8 AU, respectively; the DC values of group L showed 66.7 ± 10.9 AU, 56.1 ± 11.8 AU, 64.3 ± 11.0 AU, 56.1 ± 11.9 AU, 64.0 ± 10.9 AU and 63.5 ± 11.4 AU, respectively; and the DC values in group L were a little larger than those of group H, although the difference was not statistically significant (Table 2).

The AC values of six consecutive measurements in group H were 9.4 ± 1.1 AU, 8.5 ± 1.3 AU, 9.1 ± 1.0 AU, 8.3 ± 1.1 AU, 9.1 ± 1.2 AU and 9.6 ± 1.2 AU, respectively, and the AC values of group L were 10.3 ± 1.3 AU, 7.9 ± 1.1 AU, 9.6 ± 1.1 AU, 7.6 ± 1.0 AU, 10.0 ± 1.2 AU and 9.4 ± 1.1 AU respectively.

The PP indices of group H were 0.20 ± 0.02, 0.18 ± 0.01, 0.20 ± 0.02, 0.19 ± 0.02, 0.20 ± 0.02 and 0.20 ± 0.02, respectively, and the PP indices of group L were 0.18 ± 0.02, 0.17 ± 0.02, 0.18 ± 0.02, 0.17 ± 0.02, 0.18 ± 0.02 and 0.17 ± 0.02, respectively. The mean of PP of four relaxed states (BL, R1, R2 and R3) in group H was a little higher (0.20) than that in group L (0.18), although the difference was not significant (Table 2).

As the participants were actively stretching their gastrocnemius muscles by performing ankle dorsiflexion (AS1 and AS2), the blood perfusion decreased significantly and then recovered during the muscle relaxation periods (R1 and R2). The blood perfusion reduction and recovery between adjacent measurements were statistically significant (*p* < 0.05) in the participants with lower flexibility (group L), while the blood perfusion was relatively stable for the higher flexibility group. The standard deviation of DC values for the six consecutive measurements in group L was 4.1 AU, which was larger than 1.8 AU in group H. Not only the DC flux exhibited a larger fluctuation, the standard deviation of AC values in group L was also larger (1.0 AU in group L, 0.5 AU in group H). While the muscle stretching had a major influence on both DC flux and AC flux, the PP indices were more consistent.

After the intervention of muscle stretching, the blood perfusion in relaxed state R3 decreased related to the baseline value by 5% (from 66.7AU to 63.5 AU) in group L; whereas the decrease of perfusion was not significant in group H (from 54.0 AU to 54.0 AU).

## 4. Discussion

By using the non-invasive LDF technique, the regional flexibility of the gastrocnemius muscle was evaluated quantitatively according to the microcirculation characteristics during different muscle stretching and relaxed states. Even the LDF signals obtained from non-invasive technique with a skin probe revealed the blood perfusion waveform without a definite period and therefore it was difficult to determine the period of each perfusion pulse, a mean perfusion waveform in one heartbeat interval (Figure 4) could be derived by the modified beat-to-beat algorithm. Three indices of DC (the mean blood flow), AC (pulsatile blood flow) and PP (AC to DC ratio) were then defined according to this mean perfusion waveform. As shown in Figure 5a,b, the microcirculatory perfusion remained steadily in individuals with higher muscle flexibility along the six consecutive muscle stretching and relaxed states, whereas the perfusion declined with muscle stretching in the lower flexibility group.

Along with the six measurements, both the conventional DC flux and AC flux fluctuated; they decreased in active muscle stretching, and recovered in muscle relaxed. Interestingly, the participants in group H showed a more stable blood perfusion than group L did. Consequently, the stability (e.g., standard deviation) might be a feasible indicator of muscle flexibility, while a stable microcirculation in muscle stretching reflected higher muscle flexibility. The results were consistent with Otsuki's finding on the tibialis anterior muscle with passive muscle stretching by using NIRS technique [9]. It was found that ballet-trained subjects (*i.e*., with high muscle flexibility) stretched their muscles without excessive attenuation in muscle blood flow (compared with untrained people). The finding might be associated with the alterations in capillary geometry and luminal diameter consequent to increase the muscle sarcomere length during muscle stretching, while the capillary structure could be more flexible in trained subjects. According to Poole's study [10], capillary tortuosity around spinotrapezius decreased systematically with the increase of sarcomere length. When the sarcomere length increased up to 2.6 microns, most capillaries appeared to be highly oriented along the fiber longitudinal axis. Further increase in sarcomere length above this value would reduce the capillary diameter, and therefore the blood perfusion decreased. In other words, the better the muscles flexibility was; the higher stability associated with blood perfusion during the muscle length changed.

In addition to the fluctuations in DC blood flow and AC blood flow, two phenomena were found for discriminating the muscle flexibility, including a higher baseline DC value and a larger decline in DC value after the repeatedly active muscle stretching. Figure 5a shows a higher DC value in the baseline (BL) and a larger decrease from BL to R3 in the inflexible group. The inflexible subjects with a greater blood perfusion on gastrocnemius muscle in the present study might be related to the circulatory status similar to the local venous stasis or chronic venous insufficiency (CVI) in a very mild stage. It was believed that chronic venous insufficiency (CVI) of the lower extremities was a condition caused by abnormalities of the venous wall and valves that it led to obstruction or reflux of blood flow in the veins. The local accumulation of blood might lead to secondary lymphedema, and the trapped fluid will cause enlarged and tight muscle [22]. The inflexible subjects also showed a decreased DC value on gastrocnemius muscle after performing the active ankle dorsiflexion. The possible reason might be associated with the importance of ankle movement in promoting venous flow. Moloney conducted a hemodynamic study, in which the blood flow velocities generated from the voluntary calf muscle contraction, calf muscle pump, was related to a heel lift motion resulting in planter flexion and voluntary calf muscle contraction [23]. Their results emphasized the importance of ankle exercises accelerating venous return. In contrast, the ankle joint disease caused by venous hypertension or other medical conditions such as arthritis might result in reduced range of muscle flexibility around ankle joint, thereby reducing the effect of ankle joint movements in promoting venous return [24].

Among noninvasive methods for monitoring perfusion in peripheral tissues, the peripheral perfusion index (PI) derived from the photoplethysmographic signal (PPG) of pulse oximetry has been widely used in critically ill patients and neonatal care [18,19]. A lower PI value reflected the presence of poor peripheral perfusion in critically ill patients in these studies. In a similar manner, the PP index was defined as the ratio of pulsatile component (AC) to nonpulsatile component (DC) in this investigation. As a higher PI value represents better peripheral perfusion [18], participants in group H also had higher PP values, especially in relaxed states of BL, R1, R2 and R3. It implied that the peripheral perfusion of group H was better. The reason for it not reaching the statistical significance appeared on both groups, aged 20–21 years, without musculoskeletal disorders in lower extremities, with 3 h of physical education class each week. In this case, although PP indices were more stable and could not represent muscle flexibility as the fluctuations of the DC and AC values did, it could provide the information of the perfusion difference between groups.

Fry combined ultrasound imaging with motion analysis technology to measure the distances between remote anatomical landmarks [25]. The length of the belly of the medial gastrocnemius muscle was estimated. Their landmarks were chosen because the easy identification under ultrasound scanning was straightforward. However, their proximal end of the gastrocnemius was difficult to visualize because of the complex of overlapping muscle and tendon that crossed the posterior aspect of the knee joint. The present study measuring the microcirculation within the active muscle contraction range may reflect the clinical measurement of active RoM without too much surrounding non-contractile tissues interfering the joint motion.

Near infrared spectroscopy and LDF are two of the most popular techniques applied to the assessment of tissue physiology. LDF techniques provide the direct measurement of microcirculatory perfusion, while near infrared spectroscopy measures the oxygenation, but not the blood flow [26]. The laser-Doppler method was first proposed by Öberg [27,28] and then elaborated for intramuscular application by Salerud [29]. Røe has extensively studied the reliability of blood flux measurements by intramuscular LDF technique and found that large variations between measurement sites (because of small monitored tissue volume, 1 mm^3^), between repeated contractions and between measurement days should be concerned [30] in spite that the reliabilities during rest and muscle contractions were good. Besides, the invasive LDF technique might also interfere in the circulation measured. In comparison with intramuscular techniques, the non-invasive technique using a higher laser power and a probe with wider fiber separation of 4 mm were applied in the present research. With the lower influence on the circulation, the reliabilities of measurements during 1-min baseline were high for both groups. A larger sampled volume also further reduced the variation due to the heterogeneity of muscle blood flow. Therefore, the inter-rater reliability was also high. These advantages improve the practicability of LDF.

Although the sampling depth of the typical non-invasive LDF technique was limited, a higher laser power and a wider probe increase the sampling depth. According to the investigation using the same model, which provided the estimation of the measurement depth and volume in LDF based on the Doppler scattering events simulated with the Monte Carlo technique, there was a 65.0% of photon sampling depths greater than 1.4 mm [14]. Moreover, the related research found that the deeper macrocirculatory response could likely affect the microcirculation (including capillaries, arterioles, and venules) upon them [31]. The present findings warranted further investigation for the consistence of the measurements determined by non-invasive and invasive LDF techniques, and therefore the present technique could be applied for use in the applications of flexibility assessment.

## 5. Conclusions

By analyzing perfusion signals using a beat-to-beat algorithm, the higher muscle flexibility is associated with the better stability of microcirculatory perfusion under the intervention of muscle stretching. With the advantages of non-invasiveness, quantification, and easy use, the primary findings of the study reveal that the microcirculatory characteristics determined by using a non-invasive LDF could be developed as an objective tool for the assessment of muscle flexibility.

## Figures and Tables

**Table 1.** Characteristics of the subjects grouped by Active RoM of ankle dorsiflexion, expressed in mean (SD).

	**Higher Flexibility Group** **(Group H) *n* = 15**	**Lower Flexibility Group** **(Group L) *n* = 15**	***p* Value**
Weight (kg)	53.7 ± 9.4	59.4 ± 9.8	0.12
Height (cm)	165.7 ± 7.3	167.8 ± 8.2	0.47
BMI	19.5 ± 2.7	21.0 ± 2.4	0.13

Active RoM of ankle dorsiflexion (degree)	20.3 ± 4.2	11.0 ± 3.6	<0.0001

**Table 2.** The p-value of inter-group variation.

	**BL**	**AS1**	**R1**	**AS2**	**R2**	**R3**
**DC**	0.3483	0.7195	0.4514	0.6363	0.4027	0.4995
**AC**	0.6206	0.7444	0.7733	0.6519	0.6039	0.9333
**PP**	0.4160	0.7554	0.5153	0.5383	0.4643	0.3671

## References

[1] Vanhees L., Lefevre J., Philippaerts R., Martens M., Huygens W., Troosters T., Beunen G. (2005). How to assess physical activity? How to assess physical fitness?. Eur. J. Cardiovasc. Prev. Rehabil..

[2] Caspersen C.J., Powell K.E., Christenson G.M. (1985). Physical activity, exercise, and physical fitness: Definitions and distinctions for health-related research. Pub. Health Rep..

[3] Wilder R.P., Sethi S. (2004). Overuse injuries: Tendinopathies, stress fractures, compartment syndrome, and shin splints. Clin. Sports Med..

[4] Lun V., Meeuwisse W.H., Stergiou P., Stefanyshyn D. (2004). Relation between running injury and static lower limb alignment in recreational runners. Br. J. Sports Med..

[5] Kisner C., Colby L.A. (2007). Therapeutic Exercise: Foundations and Techniques.

[6] Page P. (2012). Current concepts in muscle stretching for exercise and rehabilitation. Int. J. Sports Phys. Ther..

[7] Moseley A.M., Crosbie J., Adams R. (2003). High- and low-ankle flexibility and motor task performance. Gait Posture.

[8] Johanson M., Baer J., Hovermale H., Phouthavong P. (2008). Subtalar joint position during gastrocnemius stretching and ankle dorsiflexion range of motion. J. Athl. Train..

[9] Otsuki A., Fujita E., Ikegawa S., Kuno-Mizumura M. (2011). Muscle oxygenation and fascicle length during passive muscle Stretching in ballet-trained subjects. Int. J. Sports Med..

[10] Poole D.C., Musch T.I., Kindig C.A. (1997). *In vivo* microvascular structural and functional consequences of muscle length changes. Am. J. Physiol Heart Circ. Physiol..

[11] Strøm V., Knardahl S., Stanghelle J.K., Røe C. (2009). Pain induced by a single simulated office-work session: Time course and association with muscle blood flux and muscle activity. Eur. J. Pain.

[12] Strøm V., Røe C., Knardahl S. (2009). Work-induced pain, trapezius blood flux, and muscle activity in workers with chronic shoulder and neck pain. Pain.

[13] Røe C., Knardahl S. (2002). Muscle activity and blood flux during standardised data-terminal work. Int. J. Ind. Ergon..

[14] Clough G., Chipperfield A., Byrne C., de Mul F., Gush R. (2009). Evaluation of a new high power, wide separation laser Doppler probe: Potential measurement of deeper tissue blood flow. Microvasc. Res..

[15] Chao P.T., Jan M.Y., Hsiu H., Hsu T.L., Wang W.K., Lin Wang Y.Y. (2006). Evaluating microcirculation by pulsatile laser Doppler signal. Phys. Med. Biol..

[16] Hsiu H., Hsu W.C., Chang S.L., Hsu C.L., Huang S.M., Lin Y.Y. (2008). Microcirculatory effect of different skin contacting pressures around the blood pressure. Physiol. Meas..

[17] Jan M.Y., Hsiu H., Hsu T.L., Wang Y.Y., Wang W.K. (2000). The importance of pulsatile microcirculation in relation to hypertension: Studying the relationship between abdominal aortic blood pressure and renal cortex flux. IEEE Eng. Med. Biol. Mag..

[18] Lima A.P., Beelen P., Bakker J. (2002). Use of a peripheral perfusion index derived from the pulse oximetry signal as a noninvasive indicator of perfusion. Crit. Care Med..

[19] He H.W., Liu D.W., Long Y., Wang X.T. (2013). The peripheral perfusion index and transcutaneous oxygen challenge test are predictive of mortality in septic patients after resuscitation. Crit. Care.

[20] Fleiss J.L., Shrout P.E. (1977). The effects of measurement errors on some multivariate procedures. Am. J. Pub. Health.

[21] Hallgren K.A. (2012). Computing inter-rater reliability for observational data: An overview and tutorial. Tutor Quant. Meth. Psychol..

[22] Berliner E., Ozbilgin B., Zarin D.A. (2003). A systematic review of pneumatic compression for treatment of chronic venous insufficiency and venous ulcers. J. Vasc. Surg..

[23] Clarke Moloney M., Lyons G.M., Breen P., Burke P.E., Grace P.A. (2006). Haemodynamic study examining the response of venous blood flow to electrical stimulation of the gastrocnemius muscle in patients with chronic venous disease. Eur. J. Vasc. Endovasc. Surg..

[24] Dix F.P., Brooke R., McCollum C.N. (2003). Venous disease is associated with an impaired range of ankle movement. Eur. J. Vasc. Endovasc. Surg..

[25] Fry N.R., Childs C.R., Eve L.C., Gough M., Robinson R.O., Shortland A.P. (2003). Accurate measurement of muscle belly length in the motion analysis laboratory: Potential for the assessment of contracture. Gait Posture.

[26] Boushel R., Langberg H., Olesen J., Gonzales-Alonzo J., Bülow J., Kjaer M. (2001). Monitoring tissue oxygen availability with near infrared spectroscopy (NIRS) in health and disease. Scand. J. Med. Sci. Sports.

[27] Oberg P.A., Nilsson G.E., Tenland T., Holmström A., Lewis D.H. (1979). Use of a new laser Doppler flowmeter for measurement of capillary blood flow in skeletal muscle after bullet wounding. Acta Chir. Scand. Suppl..

[28] Nilsson G.E., Tenland T., Oberg P.A. (1980). Evaluation of a laser doppler Flowmeter for measurement of tissue blood flow. IEEE Trans. Biomed. Eng..

[29] Salerud E.G., Oberg P.A. (1987). Single-fibre laser Doppler flowmetry. Med. Biol. Eng. Comput..

[30] Røe C., Damsgard E., Knardahl S. (2008). Reliability of bloodflux measurements from the upper trapezius muscle during muscle contractions. Eur. J. Appl. Physiol..

[31] Serov A., Lasser T. (2006). Full-field high-speed laser Doppler imaging system for blood-flow measurements. Biomed. Clin. Diagn. Syst. IV.

